# Autoimmune Progesterone Dermatitis

**DOI:** 10.7759/cureus.19217

**Published:** 2021-11-02

**Authors:** Shahmeen Irshad, Muhammad S Haider, Madiha F Master, Nasir Asif, Ambreen Khalil

**Affiliations:** 1 Internal Medicine, Richmond University Medical Center, New York, USA; 2 Medicine, Philadelphia College of Osteopathic Medicine, Philadelphia, USA; 3 Medicine, Rutgers University, Newark, USA

**Keywords:** autoimmune disease, dermatitis, allergy and immunology, erythema, progesterone

## Abstract

The condition autoimmune progesterone dermatitis (APD) is an immune disorder, observed among women, primarily due to progesterone surge during menstrual cycle. Here, we present a case of a 29-year-old female with recurrent severe skin eruptions associated with her menstrual cycle that commenced a few years ago. She presented with blistered skin lesion of the body and also blisters in oropharyngeal mucosa leading to a variety of symptoms ranging from pruritus to difficulty in swallowing. Recognition of this process is important as it can result in significant debility among women. Our patient was treated with steroids and antihistamines to provide symptomatic relief and was encouraged to resume her oral contraceptive pill, which is a more definitive therapy.

## Introduction

Autoimmune progesterone dermatitis (APD) is a rare form of hypersensitivity reaction to cyclic variation in progesterone levels in women of childbearing age [1]. The clinical presentation can range from urticaria, to stomatitis, to anaphylaxis [2-4]. Treatment is generally focused on alleviating the symptoms during each episode and continuation of oral contraceptive pills (OCPs).

## Case presentation

A 29-year-old female, brought in by ambulance, complained of blisters all over her body. The patient stated that she had a history of erythema multiforme and urticaria for the past seven years and gets a flare every time she menstruates. She was previously on oral contraceptives and noticed that her symptoms had decreased at that time. However, after she discontinued use about a year ago, skin eruptions seemed to be more severe.

**Figure 1:**
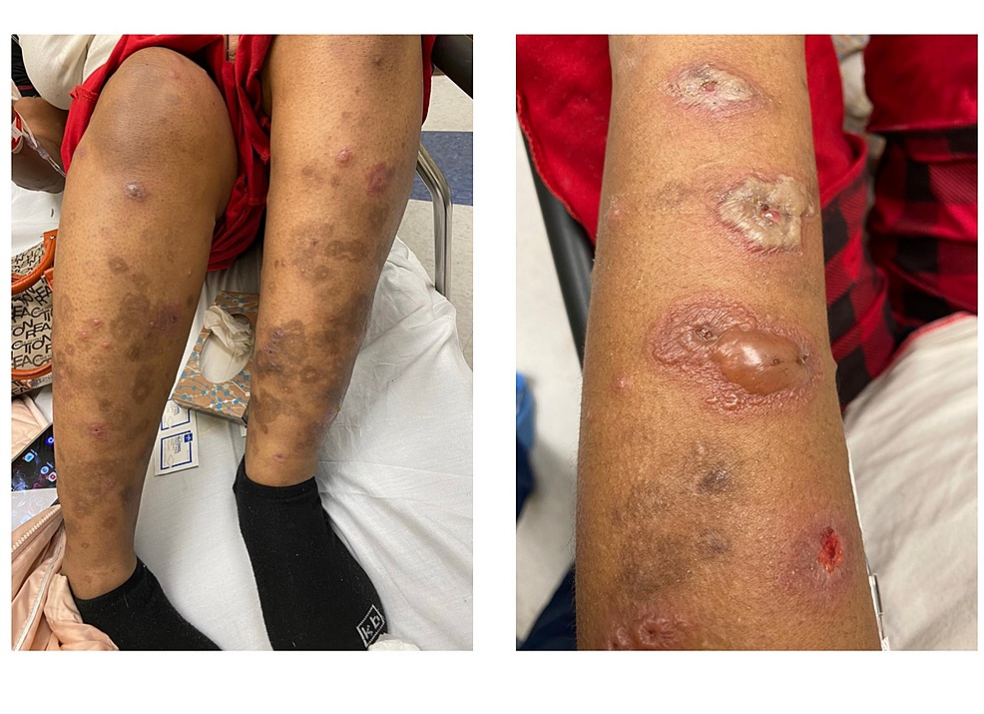
Lesions with different stages of healing

She endorsed that this episode of the flare was much worse than what she had dealt with in the past year. She stated that she had painful blisters all over her body including extremities, back, and oral mucosa for four days prior to the start of her periods (Figure 1). She then decided to come to the emergency department as the symptoms were progressively getting worse and she was unable to swallow due to pain, which she rates at 10/10 in intensity.

The patient had an initial temperature of 103.3 °F in emergency room. Other vital signs were also within normal limits. The patient's white cell count was marginally elevated during the course of hospital stay, whereas the eosinophil count remained within normal limits. Blood culture and urine analysis were normal. Hepatitis C virus (HCV), HIV, and syphilis were ruled out as well.

The patient was primarily treated with intravenous steroids and diphenhydramine. After receiving a loading dose of 125mg IV Solu-Medrol, she was started on both IV Solu-Medrol 60mg and diphenhydramine 25mg twice a day. On the second day of her hospital day, she was started on topical clobetasol as well.

In the following few days, she continued to feel severe pain which was only partially alleviated by morphine and continued to have new lesions in oral cavity and lower extremities. She was unable to open her mouth or eat as blisters in her oral mucosa continued to worsen. Her blisters began to easily tear, and some formed central umbilication. Airway was noted to be stable with no dyspnea or stridor. During the course of hospitalization, her blisters in the oral mucosa began to regress, allowing for her to drink liquids with minimal pain. On the sixth day of hospitalization, the patient was able to tolerate a solid diet with minimal dysphagia. Blisters on her extremities began to resolve as no inflamed bullae were noted, and the patient no longer required morphine for pain management. The patient was then discharged home with a six-week taper of steroids with topical clobetasol.

Our patient was diagnosed with autoimmune progesterone dermatitis based on history and physical examination. She was advised to resume her OCPs and followed up with obstetricians-gynecologists (OB-GYN) and dermatologist as an outpatient for long-term control of her symptoms.

## Discussion

Autoimmune progesterone dermatitis is a rare type of hypersensitivity disease recurring monthly in women during menstrual cycle. Although pathogenesis of this clinical condition is not yet clear, the major mechanism is considered to be exposure to exogenous and/or endogenous progesterone causing hypersensitivity reactions leading to clinical manifestation of this disease [5].

In 1921, the first case of APD was published in which premenstrual serum causing urticarial lesions was observed [1]. To this date, less than 200 cases of this rare condition have been reported in literature [6,7].

Currently, there is no definite age of APD onset, the earliest age a woman can experience APD is at menarche [8]. There has been an increased prevalence of APD occurring in women during the third decade of life. This was seen in our 29-year-old patient who had a seven-year history of disease flare-up coinciding with her menstruation. APD symptoms, especially skin lesions, usually appear during the luteal phase when there is a spike in the level of progesterone level, approximately 3-10 days before the start of the menstrual cycle [6,7]. Resolution of symptoms usually occurs a few days after the onset of menses.

The clinical features of autoimmune progesterone dermatitis can include both cutaneous and noncutaneous symptoms. The most common clinical signs include urticaria, erythema multiforme, angioedema, Stevens-Johnson-like syndrome, and eczema [9,10]. Noncutaneous manifestations can include asthma [6] and severe anaphylaxis reaction [4]. Our patient described her current symptoms as the worst flare yet, with erythema multiforme including severe mucosal involvement leading to odynophagia.

Additionally, symptoms of APD can also occur during pregnancy. The disease manifestation might improve due to the spike of maternal progesterone levels during pregnancy. This increase in progesterone can decrease the symptoms of APD by acting as a desensitizing agent [4]. Therefore, progesterone desensitization therapy is another useful therapy to be used in patients failing standard medication therapy or who do not wish to be on long-term steroids [6].

Clinical history and physical examination are the primary way to diagnose APD. Diagnostic criteria proposed by Warin et al. [11] include lesions on the skin accompanied with onset of menstrual cycle, a positive intradermal test, and improvement of symptoms after treatment with progesterone inhibition therapies. The intradermal test can support the diagnosis of APD based on the symptoms; on the contrary, in asymptomatic women, a positive intradermal test can result in false negative; therefore, this test might not be the most efficient diagnostic criteria for diagnosis of APD.

APD is mainly treated with ovulation suppression. Primary treatment includes prescribing a combination of oral contraceptives. Other successful treatment options include use of gonadotropin-releasing hormone (GnRH) agonists, danazol and tamoxifen; topical and oral antihistamines; steroids to treat cutaneous symptoms; and bilateral oophorectomy in patients experiencing persistent symptoms of APD [4]. 

## Conclusions

Autoimmune progesterone dermatitis is a rare condition occurring in response to exogenous or endogenous progesterone. The case highlights the severe nature of symptoms that progesterone hypersensitivity can present with. It is therefore important that clinicians working both in outpatient and inpatient setups are able to correctly identify this rare condition based upon taking detailed history and conducting physical examinations as well as educating patients about the pathophysiology of their symptoms so they can better deal with this recurrent condition. Recognition of this rare condition needs a high index of suspicion. Treatment is generally done in collaboration with gynecology.
